# Velo-Palatine Tumor

**Published:** 1914-11

**Authors:** 


**Velo-Palatine Tumor.** Progress Med., p. 288, June 13, 1914, describes a fibromatous and angiomatous tumor, very dense and adherent, ocoluding one side of the vault. It was at first taken for an abscess. He considers it of branchial origin and very rare.

